# Interlaboratory Comparison of the Pneumococcal Multiplex Opsonophagocytic Assays and Their Level of Agreement for Determination of Antibody Function in Pediatric Sera

**DOI:** 10.1128/mSphere.00070-18

**Published:** 2018-04-25

**Authors:** A. Balloch, L. Roalfe, N. Ekstrom, C. D. Nguyen, L. Spry, R. A. Marimla, P. V. Licciardi, D. Goldblatt, E. K. Mulholland

**Affiliations:** a Pneumococcal Immunology Laboratory, Murdoch Children’s Research Institute, Melbourne, Australia; b UCL Great Ormond Street Institute of Child Health, London, United Kingdom; c The National Institute for Health and Welfare, Helsinki, Finland; d Department of Paediatrics, University of Melbourne, Melbourne, Australia; e London School of Hygiene and Tropical Medicine, London, United Kingdom; Food and Drug Administration

**Keywords:** immunization, MOPA, pneumococcal IgG

## Abstract

Opsonophagocytic assays are used to measure functional antibodies important in protection against pneumococcal capsular antigens. There have been efforts to standardize these methods, as the assays are commonly used to measure vaccine immunogenicity. We report here the results from three international laboratories using their own methods, based on the recommended WHO standard method. We tested 30 pediatric sera, before and after administration of a 13-valent conjugate pneumococcal vaccine, against all 13 serotypes. The three laboratories demonstrated good agreement using their own standardized multiplex opsonophagocytosis assay protocols, particularly postimmunization for those serotypes in the vaccine. While serotype-specific IgG methods have already been internationally standardized and are currently used as a measure of vaccine immunogenicity, this report demonstrates that despite minor differences in methods and a minor variation in response to nonvaccine serotypes, the results from opsonophagocytic assays across the three laboratories may be compared with confidence.

**IMPORTANCE** When measuring a functional antibody response to pneumococcal immunization, it is imperative that a specific, reproducible, accurate, and standardized assay with acceptable inter- and intra-assay variation be advocated internationally to allow for meaningful comparison of results between laboratories. We report here the results of a collaboration between 3 international laboratories testing 30 pediatric samples against the 13 serotypes in Prevenar13.

## Abstract (precis)

When measuring a functional antibody response to pneumococcal immunization, it is imperative that a specific, reproducible, accurate, and standardized assay with acceptable inter- and intra-assay variation be advocated internationally to allow for meaningful comparison of results between laboratories. We report here the results of a collaboration between 3 international laboratories testing 30 pediatric samples against the 13 serotypes in Prevenar13.

## INTRODUCTION

Pneumonia remains a leading cause of infant mortality in children under 5 years of age, with almost 1.3 million deaths in 2011 (1). One vaccine-preventable cause of severe pneumonia, Streptococcus pneumoniae (pneumococcus), causes at least 18% of severe episodes and 33% of deaths worldwide, with the majority of episodes occurring in the low- and middle-income countries (2). Data from December 2015 show that pneumococcal immunization programs have been introduced into 129 countries, with global coverage estimated at 37% (3). The majority of pneumococcal vaccine trials report serotype-specific IgG as a primary measure of vaccine immunogenicity (4); however, measurement of antibody function provides more relevant information in terms of host protection.

Antibody-mediated killing of S. pneumoniae by phagocytes, known as opsonophagocytosis, is an important mechanism of host protection against pneumococcal infections. Opsonophagocytic assays (OPAs) have been developed to evaluate pneumococcal vaccine immunogenicity by mimicking *in vivo* opsonophagocytosis.

Guidelines for the measurement of functional serotype-specific pneumococcal antibodies are detailed in the World Health Organization document *Protocol for Multiplexed Opsonophagocytic Killing Assay (UAB-MOPA) for Antibodies against* Streptococcus pneumoniae (5). However, laboratories use a variety of methods, often with minor modifications, which may result in varied results. It is therefore imperative that a specific, reproducible, accurate, and standardized assay with acceptable inter- and intra-assay variation be advocated internationally to allow for meaningful comparison of results between laboratories.

We report here a bridging exercise undertaken in 2014 and 2015 between three international pneumococcal research laboratories. For the purposes of this report, the laboratories have been deidentified. Each laboratory used its own standardized MOPA protocols and reagents to test the common pediatric serum sample panel. This is the first multilaboratory study comparing MOPAs with pediatric samples.

## RESULTS

### OPAs.

Laboratories A and B provided results for all samples. Laboratory C was unable to report the results for one sample for serotype 1, nine samples for serotype 3, two samples for serotype 6A, one sample for serotype 18C, and three samples for serotype 19A due to the samples not passing their laboratory assay criteria of a maximum killing percentage of ≤40% or ≥70% (refer to Table S1 in the supplemental material). A negative titer (<8) was recorded as 4 and was included in all statistical analyses. The geometric mean opsonic index (GMOI) and 95% confidence intervals (CI) of results submitted by each laboratory are detailed in Table 1. Refer to Tables S2 to S4 for the GMOI and 95% CI for sample 1, sample 2, and sample 3.

10.1128/mSphere.00070-18.4 TABLE S1  Numbers of samples with negative results (OI < 8) or missing results for each of the three laboratories. Download TABLE S1, DOCX file, 0.02 MB. Copyright © 2018 Balloch et al. 2018 Balloch et al. This content is distributed under the terms of the Creative Commons Attribution 4.0 International license.

10.1128/mSphere.00070-18.5 TABLE S2  Geometric mean concentration (GMC), geometric mean opsonic index (GMOI), and 95% confidence intervals (95% CI) of blood sample 1 serotype-specific IgG and OI from the three laboratories. Lab A and lab B supplied results for all serotypes for the 10 samples. Lab C was unable to provide results for three samples for serotype 3, two samples for serotype 6A, and two samples for serotype 19A. Download TABLE S2, DOCX file, 0.02 MB. Copyright © 2018 Balloch et al. 2018 Balloch et al. This content is distributed under the terms of the Creative Commons Attribution 4.0 International license.

10.1128/mSphere.00070-18.6 TABLE S3  GMC, GMOI, and 95% CI of blood sample 2 serotype-specific IgG and OI from the three laboratories. Lab A and lab B supplied results for all serotypes for the 10 samples. Lab C was unable to provide results for five samples for serotype 3, one sample for serotype 18C, and one sample for serotype 19A. Download TABLE S3, DOCX file, 0.02 MB. Copyright © 2018 Balloch et al. 2018 Balloch et al. This content is distributed under the terms of the Creative Commons Attribution 4.0 International license.

10.1128/mSphere.00070-18.7 TABLE S4  GMC, GMOI, and 95% CI of blood sample 3 serotype-specific IgG and OI from the three laboratories. Lab A and lab B supplied results for all serotypes for the 10 samples. Lab C was unable to provide results for one sample for serotype 1 and one sample for serotype 3. Download TABLE S4, DOCX file, 0.02 MB. Copyright © 2018 Balloch et al. 2018 Balloch et al. This content is distributed under the terms of the Creative Commons Attribution 4.0 International license.

**TABLE 1:** GMOI and 95% CI of OIs from labs A, B, and C for study samples

Vaccine(s)	Serotype	Lab A GMOI (95% CI); no. of samples	Lab B GMOI (95% CI); no. of samples	Lab C GMOI (95% CI); no. of samples
PCV7	4	375 (148–949); 30	1,144 (408–3,206); 30	908 (374–2,204); 30
	6B	1,434 (578–3,558); 30	10,798 (4,664–24,996); 30	4,134 (1,841– 9,285); 30
	9V	862 (446–1,663); 30	15,262 (7,226–32,234); 30	1,890 (1,083–3,300); 30
	14	522 (191–1,425); 30	11,100 (6,680–18,445); 30	3,158 (1,923–5,184); 30
	18C	417 (162–1,077); 30	937 (308–2,849); 30	411 (142–1,195); 29
	19F	664 (321–1,372); 30	4,719 (2,146–10,376); 30	1,603 (852–3,017); 30
	23F	397 (189–838); 30	4,024 (2,114–7,659); 30	1,456 (729–2,911); 30

23vPPV and PCV13	1	8 (4–15); 30	27 (10–73); 30	9 (4–18); 29
	3	13 (7–22); 30	76 (34–168); 30	54 (25–117); 21
	5	16 (8–35); 30	43 (17–107); 30	17 (7–38); 30
	6A	103 (30–352); 30	1,794 (496–6,494); 30	864 (237–3,144); 28
	7F	196 (55–698); 30	14,441 (8,696–23,981); 30	2,634 (1,183–5,867); 30
	19A	64 (27–153); 30	670 (245–1,832); 30	353 (134–931); 27

### Scatterplots and Lin’s CCCs.

**FIG 1:**
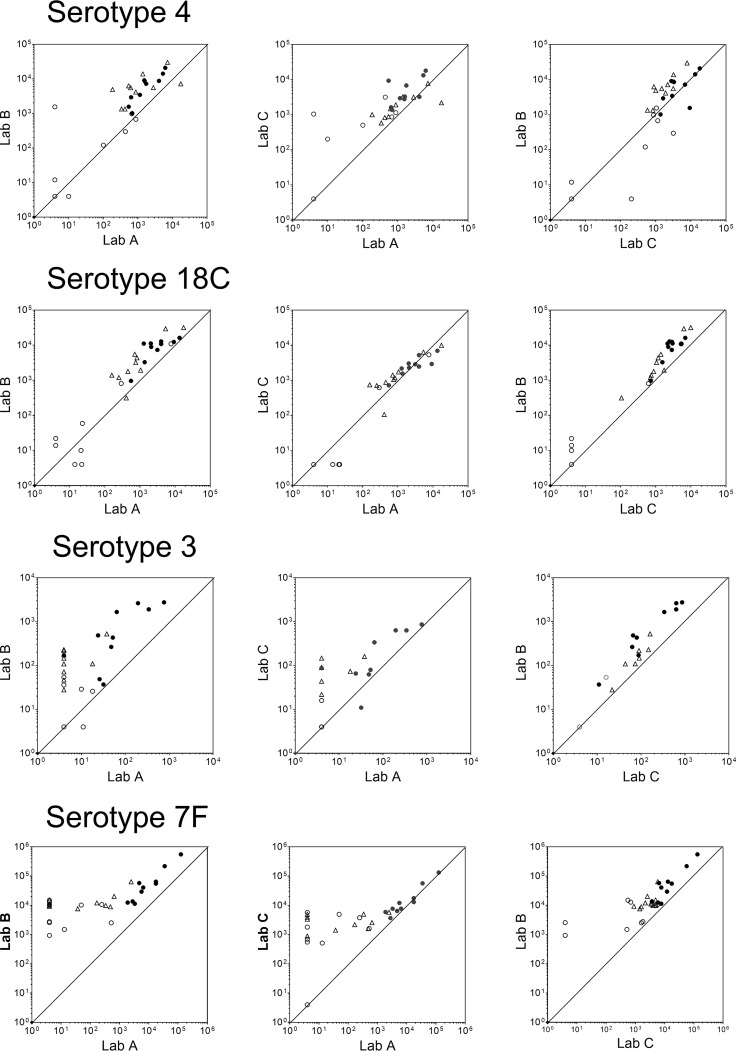
Opsonic index for lab A versus lab B, lab A versus lab C, and lab B versus lab C for serotypes 4, 18C, 3, and 7F, with triangles representing sample 1, open circles representing sample 2, and black circles representing sample 3.

Figure 1 displays representative scatterplots of the OIs for sample 1, sample 2, and sample 3 when each laboratory’s results are plotted against the other. For the two serotypes in Prevnar (a 7-valent pneumococcal conjugate vaccine [PCV7]), serotypes 4 and 18C, agreement was good, and in general, the points cluster around the diagonal line of agreement. Lin’s concordance correlation coefficient (CCC) supports this agreement; for serotype 4, in lab A and lab B, the CCC was 0.80, in lab A and lab C, the CCC was 0.80, in lab B and lab C, the CCC was 0.88 (also for serotype 18C), in lab A and lab C, the CCC was 0.95, in lab A and lab B, the CCC was 0.89, and in lab B and lab C, the CCC was 0.94 (Table 2).

**TABLE 2:** Lin’s CCCs with 95% CI for pairwise comparisons and ICCs with 95% CI^a^

Vaccine(s)	Serotype	Lin’s concordance coefficient (95% CI) [no. of pairwise comparisons]			Intraclass correlation coefficient (95% CI) [no. of pairwise comparisons]
		Lab A and lab B	Lab A and lab C	Lab B and lab C	
PCV7	4	0.80 (0.64, 0.89)	0.80 (0.64, 0.90)	0.88 (0.77, 0.94)	0.87 (0.78, 0.93)
	6B	0.64 (0.46, 0.77)	0.83 (0.71, 0.91)	0.86 (0.76, 0.92)	0.92 (0.85, 0.95)
	9V	0.28 (0.11, 0.43)	0.67 (0.45, 0.81)	0.49 (0.31, 0.63)	0.73 (0.57, 0.84)
	14	0.29 (0.15, 0.42)	0.45 (0.27, 0.60)	0.60 (0.42, 0.74)	0.65 (0.47, 0.80)
	18C	0.89 (0.80, 0.94)	0.95 (0.90, 0.97) [29]	0.94 (0.88, 0.96) [29]	0.95 (0.92, 0.98) [29]
	19F	0.61 (0.44, 0.74)	0.78 (0.63, 0.88)	0.82 (0.71, 0.89)	0.91 (0.84, 0.95)
	23F	0.36 (0.16, 0.53)	0.66 (0.46, 0.80)	0.72 (0.54, 0.84)	0.77 (0.63, 0.87)
23vPPV and PCV13	1	0.63 (0.44, 0.77)	0.98 (0.96, 0.99) [29]	0.69 (0.50, 0.82) [29]	0.79 (0.66, 0.88) [29]
	3	0.43 (0.21, 0.60)	0.57 (0.28, 0.76) [21]	0.86 (0.75, 0.93) [21]	0.73 (0.57, 0.85) [21]
	5	0.79 (0.64, 0.89)	0.96 (0.93, 0.98)	0.83 (0.69, 0.91)	0.90 (0.83, 0.95)
	6A	0.55 (0.33, 0.71)	0.65 (0.42, 0.80) [28]	0.92 (0.85, 0.96) [28]	0.83 (0.71, 0.90) [28]
	7F	0.21 (0.10, 0.31)	0.42 (0.21, 0.59)	0.48 (0.29, 0.64)	0.58 (0.39, 0.75)
	19A	0.58 (0.39, 0.72)	0.69 (0.49, 0.83) [27]	0.88 (0.77, 0.93) [27]	0.88 (0.79, 0.93) [27]

a Lab A and lab B supplied results for all serotypes for the 30 samples. Lab C was unable to provide results for one sample for serotype 1, nine samples for serotype 3, two samples for serotype 6A, one sample for serotype 18C, and three samples for serotype 19A. The numbers in brackets are the numbers of pairwise comparisons for the incomplete data sets.

For the two non-PCV7 serotypes, 3 and 7F, there was greater discordance. For serotype 3, 53% of samples tested by lab A were below the limit of detection, while this percentage was 19% or 20% for lab C or lab B, respectively. In the lab A paired scatterplots for serotype 3, this is clearly seen with the majority of matched titers from both lab B and lab C above the diagonal line of agreement. For a serotype 3 comparison between lab B and lab C, Lin’s concordance data support a good concordance of 0.86; however, the scatterplot demonstrates that the OIs reported by lab B were higher overall. This was also seen for serotype 7F, with lab A reporting a negative titer for 33% (10 of 30) of samples and lab C reporting a negative titer for 7% (2 of 30) of samples. Lab B reported a positive titer for all 30 samples. Scatterplots for the remaining serotypes are shown in Fig. S1 to S3.

10.1128/mSphere.00070-18.1 FIG S1  Opsonic index for lab A and lab B, with each laboratory’s results plotted against the other for all sera tested. Triangles represent sample 1, open circles represent sample 2, and black circles represent sample 3. Download FIG S1, DOCX file, 0.9 MB. Copyright © 2018 Balloch et al. 2018 Balloch et al. This content is distributed under the terms of the Creative Commons Attribution 4.0 International license.

10.1128/mSphere.00070-18.2 FIG S2  Opsonic index for lab A and lab C, with each laboratory’s results plotted against the other for all sera tested. Triangles represent sample 1, open circles represent sample 2, and black circles represent sample 3. Download FIG S2, DOCX file, 1 MB. Copyright © 2018 Balloch et al. 2018 Balloch et al. This content is distributed under the terms of the Creative Commons Attribution 4.0 International license.

10.1128/mSphere.00070-18.3 FIG S3  Opsonic index for lab B and lab C, with each laboratory’s results plotted against the other for all sera tested. Triangles represent sample 1, open circles represent sample 2, and black circles represent sample 3. Download FIG S3, DOCX file, 0.9 MB. Copyright © 2018 Balloch et al. 2018 Balloch et al. This content is distributed under the terms of the Creative Commons Attribution 4.0 International license.

Overall, Lin’s concordance coefficients indicate a moderate-to-high agreement between lab A and lab C. The concordance between lab A and lab C for pooled OI results was 0.79 (95% CI, 0.75 to 0.83), while serotype-specific coefficients ranged between 0.42 (serotype 7F) and 0.98 (serotype 1). The agreement was serotype dependent, with highest agreement for serotypes 1, 5, and 18C and lowest agreement for serotypes 7F and 14.

The concordance between lab A and lab B for all serotypes combined was 0.65 (95% CI, 0.60 to 0.69), while serotype-specific coefficients ranged between 0.21 (serotype 7F) and 0.89 (serotype 18C). The agreement was also serotype dependent, with highest agreement for serotypes 4, 5, and 18C and lowest agreement for serotypes 7F and 9V.

The concordance between lab B and lab C for all serotypes combined was 0.88 (95% CI, 0.86 to 0.90), while serotype-specific coefficients ranged between 0.48 (serotype 7F) and 0.94 (serotype 18C). The agreement was serotype dependent, with highest agreement for serotypes 6A, 18C, and 4 and lowest agreement for serotypes 7F and 9V.

Figures S1 to S3 demonstrate scatterplots between the three laboratories, which show good agreement for most serotypes after injection of the 13-valent pneumococcal conjugate vaccine (PCV13), with the points clustering around the diagonal line. However, for serotypes 7F and 14, the agreement was weak, as shown by Lin’s concordance coefficients (for serotype 7F, the lab A and lab C CCC was 0.42, for lab A and lab B, the CCC was 0.21, and for lab B and lab C, the CCC was 0.48; for serotype 14, the lab A and lab C CCC was 0.45, the lab A and lab B CCC was 0.29, and the lab B and lab C CCC was 0.60) (Tables 3 to 5).

**TABLE 3:** Agreement in classification of OIs by labs A and B as positive or negative^a^

Lab A OI classification	No. of samples with lab B OI classification:		Total
	Negative (OI < 8)	Positive (OI ≥ 8)	
Negative (OI < 8)	48	59	107
Positive (OI ≥ 8)	6	277	283
Total	54	336	390

a Two-by-two contingency table and kappa statistics for all samples between lab A and lab B. Agreement = 83.3%; kappa = 0.51.

**TABLE 4:** Agreement in classification of OIs by labs A and C as positive or negative^a^

Lab A OI classification	No. of samples with lab C OI classification:		Total
	Negative (OI < 8)	Positive (OI ≥ 8)	
Negative (OI < 8)	68	28	96
Positive (OI ≥ 8)	8	270	278
Total	76	298	374

a Two-by-two contingency table and kappa statistics for all samples between lab A and lab C. Agreement = 90.4%; kappa = 0.73.

**TABLE 5:** Agreement in classification of OIs by labs B and C as positive or negative^a^

Lab C OI classification	No. of samples with lab B OI classification:		Total
	Negative (OI < 8)	Positive (OI ≥ 8)	
Negative (OI < 8)	50	26	76
Positive (OI ≥ 8)	1	297	298
Total	51	323	374

a Two-by-two contingency table and kappa statistics for all samples between lab B and lab C. Agreement = 92.8%; kappa = 0.75.

### Opsonic index and serotype-specific IgG.

At the time of sample 1, all children were aged 18 months and had received 2 or 3 infant doses of PCV7. The samples were taken 1 month after a reduced (20%) dose of 23-valent Pneumovax (23vPPV). The serotype-specific IgG geometric mean concentration (GMC) for all PCV7 serotypes was greater than 2.06 µg/ml, and the three laboratories reported a positive titer (OI ≥ 8) for all 7 serotypes. The serotype-specific IgG GMC and corresponding OI for the non-PCV7 serotypes were substantially lower (Table S2).

For sample 2, the serotype-specific IgG GMC for 8 of 12 serotypes (6 of 7 PCV7 serotypes) had waned significantly (*P* < 0.05) and was reflected by lower OIs for the majority of serotypes, as measured by the three laboratories (Table S3).

One month after the PCV13 booster (sample 3), the serotype-specific IgG GMCs for all PCV13 serotypes had increased significantly (*P* < 0.03), and the OIs reported by the three laboratories were all positive (≥8) for PCV7 serotypes as well as serotypes 6A, 7F, and 19A. The OIs were <8 for six samples, namely, five samples for serotype 1 and one sample for serotype 3 from lab A. OIs were also <8 for four samples for serotype 1 and two samples for serotype 5 from lab C and one sample for serotype 1 from lab B (Table S4).

### ICCs.

Intraclass correlation coefficients (ICCs) were used to examine agreement across all three laboratories (Table 2). The ICCs ranged between 0.58 and 0.95 (median ICC = 0.83), indicating moderate-to-high agreement between laboratories. Again, similar patterns of results emerged, with high ICC values for serotypes 18C, 6B, and 19F and lower ICCs for serotypes 7F and 14.

Comparison of samples with positive (titer, ≥8) and negative (titer, <8) OPA results between laboratories resulted in the following. All three laboratories recorded a negative titer as <8. Kappa coefficients were used to assess agreement in the classification of titers as positive or negative (Tables 3 to 5). We calculated overall kappa coefficients for all serotypes combined, as we were unable to compute serotype-specific kappa coefficients due to responses falling into too few categories. The overall agreement was 92.8% for lab B and lab C, with a kappa coefficient of 0.75. This indicates good agreement between lab B and lab C in terms of positive or negative classification. Agreement was slightly lower for comparisons between lab A and lab C (agreement = 90.4%, kappa = 0.73) and lower for comparisons between lab A and lab B (agreement = 83.3%, kappa = 0.51).

## DISCUSSION

This paper describes the results from three independent laboratories measuring pneumococcal opsonophagocytic responses in pediatric sera following PCV7 immunization and in the same children before and after PCV13 immunization. For the results from vaccination studies to be compared internationally, clinicians, laboratories, and pharmaceutical companies must have confidence that the assays provide reproducible and meaningful results. Here we describe the results from three laboratories, Murdoch Children’s Research Institute’s Pneumococcal Immunology Laboratory (MCRI; Australia), the UCL Great Ormond Street Institute of Child Health (UCL; United Kingdom), and the National Institute for Health and Welfare Vaccine Immunology Laboratory (Terveyden ja Hyvinvoinnin Laitos [THL]; Finland), which were provided with 30 pediatric serum samples and used their individual methods to measure the opsonophagocytic antibody responses to the 13 serotypes in PCV13.

The pneumococcal opsonophagocytic assay (OPA) was first introduced by Romero-Steiner et al. in 1997 to measure the functional activities of antibodies in serum (6). In 2003, five laboratories performing OPAs took part in a multilaboratory evaluation (7). Each laboratory was provided with the same protocol, seven target pneumococcal strains (serotypes 4, 6B, 9V, 14, 18C, 19F, and 23F), two quality control sera, and 12 paired sera from adult donors who received one dose of 23vPPV. The report concluded that the assay could be done in multiple laboratories with a high degree of accuracy. Then in 2011, six laboratories (the original five plus one) assessed agreement using their own optimized protocols (8). Five of six laboratories assayed 24 reference specimens, and one laboratory assayed 19 unique samples. The sample profile initially included 3 samples from patients preimmunized with 23vPPV and 21 samples from patients postimmunized with 23vPPV, with three preimmunization samples removed from the analysis, as the majority of the assayed values were at or less than the minimum measurable titer. The final number of samples included in the analysis was 16 samples from healthy adults who had received 23vPPV. Five laboratories tested for at least 12 of the 13 serotypes in PCV13 (one laboratory failed to report titers for serotype 3), and one laboratory tested for the 7 serotypes in PCV7. The precision, accuracy, and concordance between the postimmunization samples included in the analysis demonstrated an acceptable agreement.

Our study is the first to perform an interlaboratory comparison using pediatric samples. This is important since evaluation of PCV immunogenicity is critical following immunization during infancy. The critical components of an OPA are the pneumococcal strain used, the preparation and encapsulation of the serotype, the source of the complement, heat inactivation of the sera, and the cultured phagocytes. The method differences between the laboratories were minimal (Table 6). Each laboratory used identical organisms from BEI Resources (with differences in the methods used for culturing the strains and subtle differences in the MOPA groupings), the same source for the baby rabbit complement, and the same phagocytic cells, with similar levels of differentiation and passaging steps. Therefore, the differences in the results obtained between the different laboratories are likely to be due to other factors, given the similar protocols used.

**TABLE 6:** Summary of the protocols used by each laboratory^a^

Lab	Pneumococcal serotypes (in multiplex or singleplex assays)	Target organisms	HL-60 differentiation protocol	HL-60 passage no. monitored?	No. of organisms/well	Acceptance criteria for organisms	HL-60 cell/ bacterium ratio	UAB or alternative assay protocol	Assay protocol reference
MCRI	Multiplex: 5, 6A, 18C, 19F Multiplex: 1, 7F, 9V, 19A Multiplex: 4, 6B, 14, 23F Singleplex: 3 (and some repeat assays)	SPEC1, OREP3, OREP4, STREP5, TREP6A, SPEC6B, OREP7F, EMC9V, STREP14, OREP18C, TREP19A, SPEC19F, EMC23F	200-ml HL-60 (2 × 10^5^ cells/ml) plus 1,550 µl DMF at 37°C and 5% CO_2_ for 5 days	Yes; after 19 or 20 passages, cells are not used in MOPA or OPA; acceptance of cells for use in OPA is based on cell viability of >70% and CD11b, CD35, and CD71 expression	2 × 10^3^ bacteria/well in MOPA; 1 × 10^3^ bacteria/well in OPA	>80% viability after freezing; storage at −80°C for no more than 6 mo; strong positive latex result; capsule integrity confirmed by Quellung; passed antibiotic specificity	400:1 in MOPA and OPA	UAB-MOPA with THL modifications; shaking platform at 220 rpm and only after addition of HL-60 cells and complement	16, 22

UCL	Multiplex: 4, 6B, 14, 23F Multiplex: 6A, 9V, 18C, 19F Multiplex: 1, 5, 7 F, 19A Multiplex: 3, 6C (6C not tested)	OREP4, SPEC6B, STREP14, EMC23F, TREP6A, EMC9V, OREP18C, SPEC19F, SPEC1, STREP5, OREP7F, TREP19A, OREP3, SPEC6C	200-ml HL-60 (4 × 10^5^ cells/ml) plus 1,600 µl DMF at 37°C and 5% CO_2_ for 5 or 6 days	Yes; generally, cells are used up to passage 25; viability, mycoplasma, CD35, and CD71 are monitored	1 × 10^4^ bacteria/well	Storage at −80°C for up to 2 yr; positive latex bead result; antibiotic sensitivity is also assessed	200:1	UAB-MOPA with UCL modifications; 10% FCS added to opsono buffer; shaking platform at 700 rpm for all incubations	5

THL	Multiplex: 5, 6A, 18C, 19F Multiplex: 1, 7F, 9V, 19A Multiplex: 4, 6B, 14, 23F Singleplex: 3, (serotype 4 for 20/30 samples)	SPEC1, OREP3, OREP4, STREP5, TREP6A, SPEC6B, OREP7F, EMC9V, STREP14, OREP18C, TREP19A, SPEC19F, EMC23F	200-ml HL-60 (2 × 10^5^ cells/ml) plus 1,550 µl DMF at 37°C and 5% CO_2_ for 5 days	Yes; acceptance of cells for use in OPA is based on cell viability of >70% and CD11b, CD35, and CD71 expression; passage no. here is <50	2 × 10^3^ bacteria/well in MOPA; 1 × 10^3^ bacteria/well in OPA	>80% viability after freezing; nonspecific killing of ≤35%; passed specificity with polysaccharide inhibition test; passed antibiotic specificity	400:1 in MOPA and OPA	UAB-MOPA with THL modifications; shaking platform at 220 rpm and only after addition of HL-60 cells and complement; no agar overlay, and TTC used only for serotype 3	23

a All organisms had been frozen. Baby rabbit complement (Pel-Freez, USA) was used for all experiments. DMF, *N*,*N*-dimethylformamide.

In the present study, lab B and lab C reported high OIs to nonvaccine serotypes. Song et al. (9) previously reported high OPA titers to serotype 7F in subjects who had received only PCV7. They concluded that the high titer might not be vaccine induced and may confer no protection in those individuals. Lin’s concordance coefficient and intraclass correlations were used to assess agreement and accuracy between the laboratories, and kappa coefficients were used to assess agreement in the classification of titers as positive or negative. Subgroup statistics to compare pre- and postimmunization samples were inappropriate, as each group comprised only 10 samples, and lab C supplied OI results for fewer samples. However, examination of the supplementary figures demonstrates a good relationship between all postimmunization (PCV13) samples and those serotypes in the vaccine. This is in agreement with the findings of Juergens et al. (10), who reported a significant correlation of serotype-specific IgG levels and OPA results for infants postvaccination with PCV13. The agreement was not as strong, especially for the non-PCV7 serotypes, in samples taken at 12 months after PCV7 vaccination and in samples taken preimmunization with PCV13 more than 4 years later (sample 1 and sample 2). This may be due to the low-titer serotype-specific IgG. Lab B and lab C detected functional responses to non-PCV7 serotypes, whereas lab A reported negative titers in those samples. The majority of immunization trials report an OI as ≥8 (positive) or <8 (negative), with no reference to the absolute serotype-specific IgG concentration. As such, it is important that the laboratories performing an OPA are consistent about measuring at this ≥8 or <8 interface. Song et al. also noted that the functionality of anti-19F antibodies was at least 10-fold lower than the functionality of antibodies to the other serotypes in PCV7. At that time, they suggested that the cutoff of ≥8 may need to be altered for each serotype. New cutoff values for both serotype-specific IgG and OPAs were published by Andrews et al. in 2014 (11), and when these cutoff values were applied to the results from the current study, the agreement between the three laboratories was even stronger. Our study demonstrates different responses to individual serotypes, with the highest OIs reported to serotypes 6B and 9V and the lowest OIs to serotype 1, 3, and 4.

It has been shown that the polysaccharide-bound antibody isotypes of IgG2 and IgG3, as well as IgM, can activate complement-mediated opsonization through the classical pathway. Thus, the measurement of serotype-specific IgG and OPAs do not always correlate. This is commonly seen in adult sera (9, 12), where nonspecific IgG with low functional capacity, as well as IgM, may be present. However, in pediatric postimmunization sera, the correlation is generally much stronger (10, 13, 14). Low levels of nonspecific IgG with low functional capacity may explain the difference in OIs measured for non-PCV7 serotypes, described in the current study for those samples after a microdose of 23vPPV and 4 years later (sample 1 and sample 2). Minor differences in methods used by the three laboratories, including the ratios of pneumococci to HL-60 cells, the storage and viability of pneumococci, the speed of the rotating mixing platform, the serotype panels used, and HL-60 differentiation protocols, may all affect the final result. This study was designed to compare the results from three independent laboratories performing assays for measurement of functional responses to pneumococcal immunization. There was no stipulation with regard to the method used, which serotypes to include in the MOPA, the growth and number of the bacteria, the growth and number of HL-60 cells, or the relative acceptance criteria (sensitivity and lower limit of measurement and uncertainty). For our current study to have maximum impact, all of these factors should be investigated further to ensure that we have confidence in the methods used in our laboratories.

A recent report from an international collaboration assigned OIs to the pneumococcal reference serum of lot 007sp and an FDA OPA calibration serum, which means that, for the first time, laboratories conducting opsonophagocytosis assays now have a designated standard to run in each of their assays (15). This will help to improve the accuracy and standardization of these assays in future studies. Furthermore, the introduction of an annual quality assurance program would be a most worthwhile exercise for all laboratories performing these assays to ensure that immunogenicity results from ongoing vaccine studies are reported with confidence.

### Conclusions.

This is the first report of an interlaboratory comparison of opsonophagocytic responses in pediatric samples. The level of agreement between the MCRI, UCL, and THL laboratories depended on the timing of the sample collection and was good for vaccine serotypes, while agreement was lower for nonvaccine serotypes. Overall, this study demonstrates good agreement in OPA measurement in pediatric samples across three laboratories, and despite minor differences in protocol, the OI results from each laboratory may be compared with confidence.

## MATERIALS AND METHODS

### Participating laboratories.

The Murdoch Children’s Research Institute Pneumococcal Immunology Laboratory (MCRI), the WHO Pneumococcal Serology Reference Laboratory, UCL Great Ormond Street Institute of Child Health, United Kingdom (UCL) and the National Institute for Health and Welfare Vaccine Immunology Laboratory, Helsinki, Finland (THL). The laboratories have been randomly deidentified, and results are reported as from lab A, lab B, and lab C.

### Samples.

Thirty sera from a sample collection stored at the MCRI were separated into three equal aliquots and were sent on dry ice to the WHO Pneumococcal Serology Reference Laboratory, UCL Institute of Child Health, United Kingdom (UCL), and the National Institute for Health and Welfare, Helsinki, Finland (THL). The samples were from 10 children who were part of the Fiji Pneumococcal Project and the Fiji follow-up study. Full details of these studies have been published previously (16, 17). Ethics approval was obtained from the Fiji National Research Ethics Review Committee (FNRERC) and the Royal Children’s Hospital Human Research Ethics Committee (Melbourne, Australia). Three samples previously collected from each study participant were used.

Five children received 3 doses of Prevnar (PCV7) at 6, 10, and 14 weeks, and 5 children received 2 doses of PCV7 at 6 and 14 weeks. At 18 months of age, all 10 children received a 20% dose of 23-valent Pneumovax (23vPPV). Sample 1 was collected at 4 weeks after 23vPPV administration, at 18 months of age. As part of the Fiji study, the guardians of these children were contacted to permit the children to be part of a follow-up study when their ages were between 4.9 and 7.4 years, and the children received a further dose of the 13-valent pneumococcal conjugate vaccine (PCV13). Sample 2 was taken prior to the PCV13 booster, and sample 3 was taken 1 month after the immunization (Table 7).

**TABLE 7:** Timing of immunization and blood collection

No. of samples (*n* = 10)	Doses and timing of PCV7 vaccination	Sample 1 patient		Median age (yr) (range) of patient providing sample 2 before PCV13 vaccination	Median no. of days (range) after PCV13 vaccination for patient providing sample 3
		Median age (yr) (range)	Median no. of days (range) after a 20% 23vPPV vaccination		
5	2 doses at 6 and 14 wk	1.47 (1.44–1.51)	30 (28–49)	6.50 (5.60–7.39)	28 (28–28)
5	3 doses at 6, 10, and 14 wk	1.44 (1.43–1.50)	29 (27–35)	5.46 (4.93–7.34)	28 (28–31)
All	2 or 3 doses	1.46 (1.43–1.51)	29 (27–49)	6.03 (4.93–7.39)	28 (28–31)

Participating laboratories were asked to include the sera in their routine assays and perform opsonophagocytosis assays (OPAs) on the serum samples using their own method for all 13 serotypes in PCV13 (1, 3, 4, 5, 6 A, 6B, 7F, 9V, 14, 18C, 19A, 19F, and 23F). The laboratories were blind to the vaccination status of the samples. Results were collated and analyzed at the MCRI.

### Strains.

The following reagents were obtained through the NIH Biodefense and Emerging Infections Research Resources Repository, NIAID, NIH: Streptococcus pneumoniae strain SPEC1, BEI Resources accession no. NR-13388; strain OREP3, NR-13389; strain OREP4, NR-13390; strain STREP5, NR-13391; strain TREP6A, NR-13392; strain SPEC6B, NR-13393; strain OREP7F, NR-13394; strain EMC9V, NR-13395; strain STREP14, NR-13396; strain OREP18C, NR-13397; strain TREP19A, NR-13398; strain SPEC19F, NR-13399; and strain EMC23F, NR-13400.

### Serotype-specific IgG.

Serotype-specific IgG was measured in the Pneumococcal Immunology Laboratory at the MCRI using a previously described modification of the gold standard WHO enzyme-linked immunosorbent assay (18).

### OPAs.

Each laboratory’s method is based on the multiplex opsonophagocytic assays (MOPAs) and OPAs developed at the University of Alabama, Birmingham, AL (5). Minor method differences between the three laboratories are summarized in Table 6. In general, sera were heat inactivated at 56°C for 30 min prior to serial dilutions in a 96-well sterile microtiter plate containing Hanks’ balanced salt solution with Mg^2+^, Ca^2+^, and gelatine (opsono buffer [OB]). Frozen stocks of pneumococci were thawed and washed with OB and diluted to 5 × 10^4^ CFU/serotype/ml. Standard bacterial dilutions were added to all wells, and the plate was incubated at room temperature for 30 min. At 30 min, 10 µl of baby rabbit complement, thawed just prior to use, was added, followed by addition of 40 µl of HL-60 cells (2 × 10^7^ cells/ml) to all test wells. A bacterial control (heat-inactivated fetal calf serum [FCS] in place of human serum and no complement) and a complement control (no serum) were included on all plates. Plates were placed on a horizontal shaker and incubated for 45 min at 37°C in 5% CO_2_. The reaction was stopped at 45 min by placing the plate on ice. A 10-µl aliquot of this mixture was then spotted onto Todd-Hewitt broth–yeast extract (THYE; 0.5%) agar plates. After application of an overlay of THYE agar containing selective antibiotics (optochin, spectinomycin, streptomycin, or trimethoprim) and 2,3,5-triphenyltetrazolium chloride (TTC), the plates were incubated overnight at 37°C in 5% CO_2_.

TTC is recommended for the detection of microbial growth by means of TTC reduction and aids in the counting of colonies using an automatic colony counter. After overnight incubation, colonies on the plates were counted, and the results were expressed as opsonization indices (OIs), where the OI is defined as the interpolated dilution of serum that kills 50% of bacteria. OIs were determined using either an in-house (THL) software or Opsotiter 3 (license granted to Licensee by UABRF) (19).

### Statistics.

Serotype-specific IgG concentrations and OIs were reported using geometric means and 95% confidence intervals. Summary statistics are presented separately by serotype and laboratory. The OIs were classified as being positive or negative based on the current recommended cutoff value of <8 (negative) and ≥8 (positive). All negative titers were assigned a value of 4 and included in all statistical analyses.

Lin’s concordance correlation coefficients (20) were used to examine agreement in OIs between each pair of laboratories (i.e., lab A versus lab B, lab A versus lab C, and lab B versus lab C). Lin’s concordance coefficient is a combined measure of accuracy (how well the data follow the 45° line of perfect agreement on a square scatterplot) and precision (how tightly the points cluster around the line). A value of 0 indicates no concordance, while a value of 1 indicates perfect concordance.

Intraclass correlation coefficients (ICCs) were used to assess agreement among the OIs across the three laboratories. The ICC values were estimated using a mixed-effects analysis of variance (ANOVA) model with a fixed effect for laboratory and a random effect for sample. The ICC is a ratio of the variance among samples to the total variance. ICC values can range between 0 and 1, with higher values representing higher correlations between measurements of the same sample.

To examine agreement in classification between the laboratories, we displayed the data using 2 by 2 contingency tables and calculated kappa coefficients, a chance-corrected measure of agreement between two categorical variables. A kappa coefficient of 0 indicates no agreement, while a value of 1 indicates perfect agreement (21). Landis and Koch (21) classification of the kappa coefficients states that 0.00 to 0.20 indicates slight agreement, 0.21 to 0.40 indicates fair agreement, 0.41 to 0.60 indicates moderate agreement, 0.61 to 0.80 indicates substantial agreement, and 0.81 to 1.00 indicates almost perfect agreement.

Due to nonnormality, OIs were log transformed, using a base 10 logarithmic transformation, prior to calculations of Lin’s concordance coefficients and ICCs. All coefficients of agreement were calculated using Stata version 14.1, while figures were produced in Stata and GraphPad Prism. A *P* of <0.05 was considered statistically significant in all cases, unless stated otherwise.

### Subgroup analyses.

*Post hoc* analysis of geometric means and 95% confidence intervals for serotype-specific IgG and OIs were calculated for each blood collection time point to investigate differences between laboratories.
