# Supplementary material for: Interlaboratory Comparison of the Pneumococcal Multiplex Opsonophagocytic Assays and Their Level of Agreement for Determination of Antibody Function in Pediatric Sera
Source: mSphere. 2018 Apr 25;3(2):e00070-18. doi: 10.1128/mSphere.00070-18 (PMC5917425; doi:10.1128/mSphere.00070-18)
Supplement: TABLE S1 [file sph002182522st1.docx]

Table S1

|  | | **Samples with negative results, frequency (%)** | | | **Samples with no results, frequency (%)** | | |
| --- | --- | --- | --- | --- | --- | --- | --- |
|  |  | **Lab A** | **Lab B** | **Lab C** | **Lab A** | **Lab B** | **Lab C** |
| PCV7 serotypes | 4 | 5 (17) | 4 (13) | 4 (13) | 0 (0) | 0 (0) | 0 (0) |
|  | 6B | 1 (3) | 1(3) | 1 (3) | 0 (0) | 0 (0) | 0 (0) |
|  | 9V | 1 (3) | 1 (3) | 1 (3) | 0 (0) | 0 (0) | 0 (0) |
|  | 14 | 5 (17) | 0 (0) | 0 (0) | 0 (0) | 0 (0) | 0 (0) |
|  | 18C | 3 (10) | 3 (10) | 7 (23) | 0 (0) | 0 (0) | 1 (3) |
|  | 19F | 1 (3) | 1 (3) | 1 (3) | 0 (0) | 0 (0) | 0 (0) |
|  | 23F | 2 (7) | 0 (0) | 2 (7) | 0 (0) | 0 (0) | 0 (0) |
| 23vPPV and PCV13 serotypes | 1 | 24 (80) | 17 (57) | 23 (77) | 0 (0) | 0 (0) | 1 (3) |
|  | 3 | 16 (53) | 6 (20) | 4 (19) | 0 (0) | 0 (0) | 9 (30) |
|  | 5 | 17 (57) | 13 (43) | 20 (67) | 0 (0) | 0 (0) | 0 (0) |
|  | 6A | 14 (47) | 5 (17) | 6 (20) | 0 (0) | 0 (0) | 2 (7) |
|  | 7F | 10 (33) | 0 (0) | 2 (7) | 0 (0) | 0 (0) | 0 (0) |
|  | 19A | 8 (27) | 3 (10) | 5 (19) | 0 (0) | 0 (0) | 3 (10) |
