# Supplementary material for: Interlaboratory Comparison of the Pneumococcal Multiplex Opsonophagocytic Assays and Their Level of Agreement for Determination of Antibody Function in Pediatric Sera
Source: mSphere. 2018 Apr 25;3(2):e00070-18. doi: 10.1128/mSphere.00070-18 (PMC5917425; doi:10.1128/mSphere.00070-18)
Supplement: TABLE S2 [file sph002182522st2.docx]

Table S2

| Serotype | | IgG  GMC (95% CI) | Lab A  GMOI (95% CI) | Lab B  GMOI (95% CI) | Lab C  GMOI (95% CI) |
| --- | --- | --- | --- | --- | --- |
| PCV7 serotypes | 4 | 3.54  (1.92, 6.51) | 1147  (412 - 3193) | 5412  (2773 - 10563) | 1711  (974 - 3006) |
|  | 6B | 11.48  (4.62, 28.50) | 1446  (247 - 8479) | 12480  (4373 - 35612) | 4452  (1632 - 12147) |
|  | 9V | 3.77  (2.02, 7.03) | 874  (506 - 1508) | 23397  (10009 - 54697) | 2586  (1629 - 4106) |
|  | 14 | 6.72  (3.13, 14.42) | 718  (248 - 2077) | 9665  (4111 - 22724) | 2611  (1270 - 5368) |
|  | 18C | 2.06  (0.97, 4.37) | 895  (327 - 2450) | 3262  (1180 - 9015) | 1251  (514 - 3040) |
|  | 19F | 19.98  (8.85, 45.14) | 1503  (476 - 4748) | 9785  (3652 - 26217) | 3377  (1581 - 7216) |
|  | 23F | 3.76  (1.73, 8.14) | 918  (344 - 2453) | 5184  (1525 - 17628) | 2130  (933 - 4861) |
| 23vPPV and PCV13 serotypes | 1 | 0.61  (0.32, 1.13) | 4  (4 - 5) | 5  (4 - 7) | 5  (3 - 9) |
|  | 3 | 1.59  (0.97, 2.61) | 6  (3 - 10) | 123  (67 - 225) | 76  (40 - 144) |
|  | 5 | 0.93  (0.48, 1.78) | 7  (3 - 13) | 35  (9 - 143) | 7  (3 - 19) |
|  | 6A | Not done | 15  (3 - 71) | 988  (133 - 7356) | 925  (122 - 7026) |
|  | 7F | 0.54  (0.25, 1.18) | 58  (9 - 354) | 13310  (8523 - 20784) | 2727  (1747 - 4255) |
|  | 19A | 3.17  (1.19, 8.46) | 26  (5 - 122) | 487  (121 - 1953) | 343  (63 - 1873) |
