# Supplementary material for: Interlaboratory Comparison of the Pneumococcal Multiplex Opsonophagocytic Assays and Their Level of Agreement for Determination of Antibody Function in Pediatric Sera
Source: mSphere. 2018 Apr 25;3(2):e00070-18. doi: 10.1128/mSphere.00070-18 (PMC5917425; doi:10.1128/mSphere.00070-18)
Supplement: TABLE S3 [file sph002182522st3.docx]

Table S3

| Serotype | | IgG  GMC (95% CI) | Lab A  GMOI (95% CI) | Lab B  GMOI (95% CI) | Lab C  GMOI (95% CI) |
| --- | --- | --- | --- | --- | --- |
| PCV7 serotypes | 4 | 0.61*  (0.27, 1.38) | 28**  (5 - 153) | 51**  (8 - 317) | 99**  (13 - 752) |
|  | 6B | 2.01**  (0.82, 4.93) | 225*  (56 - 894) | 2023*  (326 – 12573) | 745*  (142 - 3921) |
|  | 9V | 0.94**  (0.49, 1.81) | 193  (50 - 748) | 5521  (663 - 46003) | 780  (171 - 3563) |
|  | 14 | 1.40*  (0.53, 3.69) | 37*  (6 - 241) | 4816  (2156 – 10759) | 1154  (782 - 1701) |
|  | 18C | 0.35**  (0.18, 0.67) | 29  (6 - 156) | 33*  (5 – 210) | 16  (2 - 129) |
|  | 19F | 3.63**  (1.44, 9.17) | 116**  (30 - 456) | 813**  (123 – 5385) | 366**  (86 - 1555) |
|  | 23F | 1.25  (0.43, 3.61) | 81**  (15 - 423) | 2225  (497 – 9974) | 470  (74 - 3004) |
| 23vPPV and PCV13 serotypes | 1 | 0.28  (0.14, 0.58) | 4  (4 - 4) | 9  (2 - 35) | 4  (4 - 4) |
|  | 3 | 0.61*  (0.33, 1.16) | 6  (4 - 8) | 8**  (3 - 20) | 5  (2 - 11) |
|  | 5 | 0.37**  (0.27, 0.51) | 4  (4 - 4) | 4*  (4 – 4) | 4  (4 - 4) |
|  | 6A | 1.76  (0.76, 4.07) | 20  (3 - 133) | 203  (15 – 2743) | 64  (7 - 616) |
|  | 7F | 0.43  (0.21, 0.84) | 14  (4 - 55) | 4751**  (2306 - 9790) | 502**  (73 - 3435) |
|  | 19A | 2.00  (1.30, 3.09) | 16  (5 - 50) | 90  (11 - 734) | 46  (7 - 320) |

*p value < 0.05, **p value < 0.01 Wilcoxon matched-pairs signed rank test was applied to compare GMC of serotype-specific IgG Blood 1 with GMC of serotype-specific IgG Blood 2; Wilcoxon matched-pairs signed rank test was applied to compare GMOI of OI Blood 1 to OI Blood 2 from each laboratory.
