# Supplementary material for: Interlaboratory Comparison of the Pneumococcal Multiplex Opsonophagocytic Assays and Their Level of Agreement for Determination of Antibody Function in Pediatric Sera
Source: mSphere. 2018 Apr 25;3(2):e00070-18. doi: 10.1128/mSphere.00070-18 (PMC5917425; doi:10.1128/mSphere.00070-18)
Supplement: TABLE S4 [file sph002182522st4.docx]

Table S4

| Serotype | | IgG  GMC (95% CI) | Lab A  GMOI (95% CI) | Lab B  GMOI (95% CI) | Lab C  GMOIC (95% CI) |
| --- | --- | --- | --- | --- | --- |
| PCV7 serotypes | 4 | 0.61**  (0.27, 1.38) | 1660*  (884 - 3118) | 5452**  (2727 - 10902) | 4441**  (2380 - 8285) |
|  | 6B | 2.01**  (0.82, 4.93) | 9085**  (3646 - 22639) | 49858**  (18724 – 132764) | 21301**  (8824 - 51424) |
|  | 9V | 0.94**  (0.49, 1.81) | 3785**  (1897 - 7551) | 27523  (13523 - 56020) | 3349  (1697 - 6608) |
|  | 14 | 1.40**  (0.53, 3.69) | 5018**  (2081 - 12098) | 29379*  (12517 - 68956) | 10453**  (4214 - 25929) |
|  | 18 | 0.35**  (0.18, 0.67) | 2765**  (1404 - 5445) | 7679**  (4182 - 14099) | 2571**  (1649 - 4010) |
|  | 19F | 3.63*  (1.44, 9.17) | 1676**  (857 - 3276) | 13218**  (9220 – 18950) | 3331**  (2122 - 5227) |
|  | 23F | 1.25*  (0.43, 3.61) | 847*  (369 - 1945) | 5649  (2247 – 14198) | 3083*  (1603 - 5927) |
| 23vPPV and PCV13 serotypes | 1 | 0.28**  (0.14, 0.58) | 31  (5 - 177) | 406**  (66 - 2507) | 42  (5 - 334) |
|  | 3 | 0.61**  (0.33, 1.16) | 60**  (21 - 177) | 458**  (148 - 1421) | 150  (49 - 457) |
|  | 5 | 0.37**  (0.27, 0.51) | 166**  (44 - 623) | 523**  (157 – 1742) | 162**  (33 - 802) |
|  | 6A | 1.76**  (0.76, 4.07) | 3566**  (1176 - 10817) | 21926**  (6774 – 70968) | 11041**  (2657 - 45886) |
|  | 7F | 0.43**  (0.21, 0.84) | 9118**  (3598 - 23110) | 47626**  (19419 - 116804) | 13363**  (6080 - 29372) |
|  | 19A | 2.00**  (1.30, 3.09) | 667**  (355 - 1251) | 6891*  (4588 - 10352) | 2273**  (1405 - 3678) |

*p value < 0.05, Wilcoxon matched-pairs signed rank test was applied to compare GMC of serotype-specific IgG Blood 2 with GMC of serotype-specific IgG Blood 3; Wilcoxon matched-pairs signed rank test was applied to compare GMOI of OI Blood 2 to OI Blood 3 from each laboratory.
