# Supplementary material for: Interlaboratory Comparison of the Pneumococcal Multiplex Opsonophagocytic Assays and Their Level of Agreement for Determination of Antibody Function in Pediatric Sera
Source: mSphere. 2018 Apr 25;3(2):e00070-18. doi: 10.1128/mSphere.00070-18 (PMC5917425; doi:10.1128/mSphere.00070-18)
Supplement: FIG S3 [file sph002182522sf3.docx]

Fig. S3
